# Two Decades of Global Research Trends on Major Histocompatibility Complex Class I Gene on Farm Animals: Bibliometric Analysis (2001 to 2020)

**DOI:** 10.1002/vms3.70818

**Published:** 2026-02-10

**Authors:** Masixole Maswana, Dikeledi Petunia Malatji, Thobela Louis Tyasi

**Affiliations:** ^1^ Department of Agriculture and Animal Health, College of Agriculture and Environmental Sciences University of South Africa Florida South Africa; ^2^ Department of Agricultural Economics and Animal Production, School of Agricultural and Environmental Sciences University of Limpopo Sovenga South Africa

**Keywords:** disease resistance, farm animals, immunology, parasitic diseases

## Abstract

The resistance or susceptibility to various infectious and parasitic diseases is associated with the genetic polymorphisms of major histocompatibility complex Class I (MHCI) gene. The extensive practice of breeding farm animals over centuries to satisfy food demand, while largely overlooking the enhancement of disease resistance through selective breeding, has resulted in a diminished genetic capacity of these animals to combat pathogens. The study seeks to understand the global research trends using published information on MHCI gene in farm animals (cattle, goats, sheep, chicken, geese, ducks, horses and donkeys), which may identify strengths and weaknesses in research initiatives directed towards finding solutions. The Year 2014 emerged as the only year with the greatest number of publications and citations. The production output of articles over time showed that the United States of America and the United Kingdom were found to be leading in this regard. Topics that involved ‘Nucleotide sequence,’ ‘genes’ and ‘MHC Class I’ were identified to be extremely underrepresented and changing quickly. Topics with high impact but with no specialisation included ‘animals,’ ‘article,’ ‘non‐human,’ ‘female,’ ‘animal,’ ‘experiment’ and ‘bovine.’ A blend of underrepresented terms and specialised ones will enhance future topics, leading to more significant research outcomes. China and Africa must establish robust collaborations to advance MHCI studies in goats.

## Graphical abstract

In this study, a bibliometric mapping of two decades (2001–2020) of literature on the Major Histocompatibility Complex Class I (MHCI) gene in farm animals was conducted. The analysis revealed that research topics such as ‘Animals’ and ‘Major Histocompatibility Antigen Class I’ were trending, with the USA and UK contributing the highest publication output. Among farm animals, the bovine industry attracted the greatest research interest.

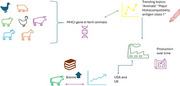

## 1 Introduction

The resistance or susceptibility to various infectious and parasitic diseases is associated with the genetic polymorphisms of Major Histocompatibility Complex Class I (MHCI) gene (Zidi et al. 2008). According to Grossen et al. (2014), the MHCI gene is responsible for gathering the information that is necessary to make farm animals susceptible to a variety of parasitic diseases. The extensive practice of breeding farm animals over centuries to satisfy food demand, while largely overlooking the enhancement of disease resistance through selective breeding, has resulted in a diminished genetic capacity of these animals to combat pathogens (Ellis and Hammond 2014).

Consequently, this has heightened reliance on pharmaceuticals and medical interventions. A study conducted by Ribeiro et al. (2021) proved to act as a conduit for scholarly exchange; this method charts the cognitive domains pertinent to farm animals of inquiry. It is crucial to discern the cooperative dynamics between authors, institutions and the prevailing research themes within the field of farm animal studies (Cui et al. 2023). Banchi et al. (2022) assert that the value of conducting a bibliometric study is found in its potential to provide insights into questions that have not been explicitly asked. The study seeks to understand the global research trends using published information on MHCI gene in farm animals (cattle, goats, sheep, chicken, geese, ducks, horses and donkeys), which may identify strengths and weaknesses in research initiatives directed towards finding solutions.

The objective of this bibliometric analysis was to uncover emerging trends related to the MHCI gene in farm animals. This review will enhance the understanding of the MHCI gene in livestock, with the anticipation that the results will assist researchers in structuring their investigative endeavours towards promising research directions in this field.

## 2 Materials and Methods

### 2.1 Data Sources

This examination was conducted using searches from the Scopus (Elsevier's data) and Web of Science (WOS) databases. Çelik. (2021) clearly states that the WOS database offers access to reference statistics of scientific publications as well as bibliographic information pertaining to these publications. Among the largest curated abstract and citation databases, Scopus provides extensive global and regional coverage of scientific journals, conference proceedings and books. It ensures that only the highest quality data is indexed through a rigorous process of content selection and re‐evaluation by an independent Content Selection and Advisory Board (Baas et al. 2020).

### 2.2 Search Strategies

The literature review employed key search terms, including ‘Major histocompatibility complex class I’ OR MHCI in conjunction with ‘farm animals’ OR cattle OR goat OR sheep OR chicken OR goose OR duck OR horse OR donkey from the years 2001 to 2020.

### 2.3 Inclusion and Exclusion Criteria

Due to the intricate nature of the research data, all relevant papers were compiled into a single dataset, with those lacking essential information being excluded. This research exclusively included book chapters, reviews, proceeding papers, abstracts and original research articles published in English, while any works that did not meet these criteria were omitted from consideration.

### 2.4 Data Extraction

The data was obtained from the Web of Science Core Collection (WoSCC) and Scopus by utilising the ‘Export Records to File’ feature along with the ‘Full Records and Cited References’ selections. The complete records were retrieved in BibTeX format to facilitate bibliometric analysis, combined using biblioshiny software, and 10 duplicated documents have been removed, moving forward with 149 documents.

### 2.5 Bibliometric Analysis

**FIGURE 1:**
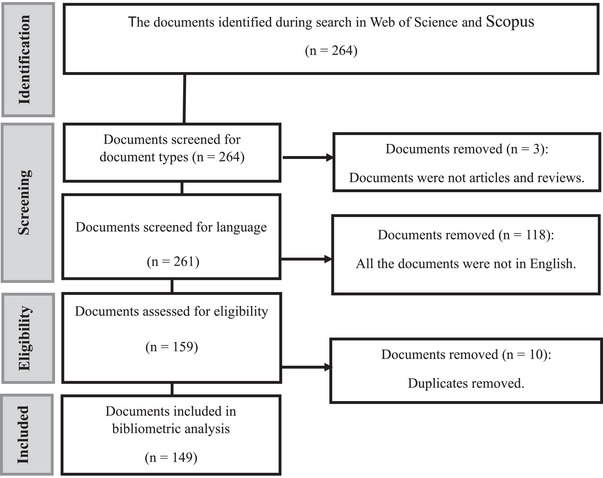
Flow chart of identification and selection of studies used in the bibliometric analysis.

The analysis was conducted utilising R Studio (version 4.3.0, 2022). In accordance with the methodology outlined by Tyasi et al. (2024), a bibliometric analysis was executed employing the Bibliometrix package within the R programming environment, focusing on 149 articles, as illustrated in Figure 1.

## 3 Results

### 3.1 Main Information

Table 1 gives overall information on the collected data. A total of 727 authors were represented, with 2.69 % international co‐authorships. Furthermore, the current study recorded an overall average of 29.06 citations per document.

**TABLE 1:** Main information.

Description	Results
Time span	2001–2020
Sources (book chapters, reviews, proceeding papers, abstracts and original research articles)	83
Documents	149
Annual growth rate %	0
Document average age	13.9
Average citations per doc	29.06
References	0
Keywords Plus (ID)	2102
Author's Keywords (DE)	2273
Authors	727
Authors of single‐authored docs	1
Single‐authored docs	1
Co‐Authors per doc	5.9
International co‐authorships %	2.69
Article	139
Article: Book chapter	1
Article: Proceedings paper	1
Book chapter	1
Review	8

### 3.2 Publication and Citation per Year

**FIGURE 2:**
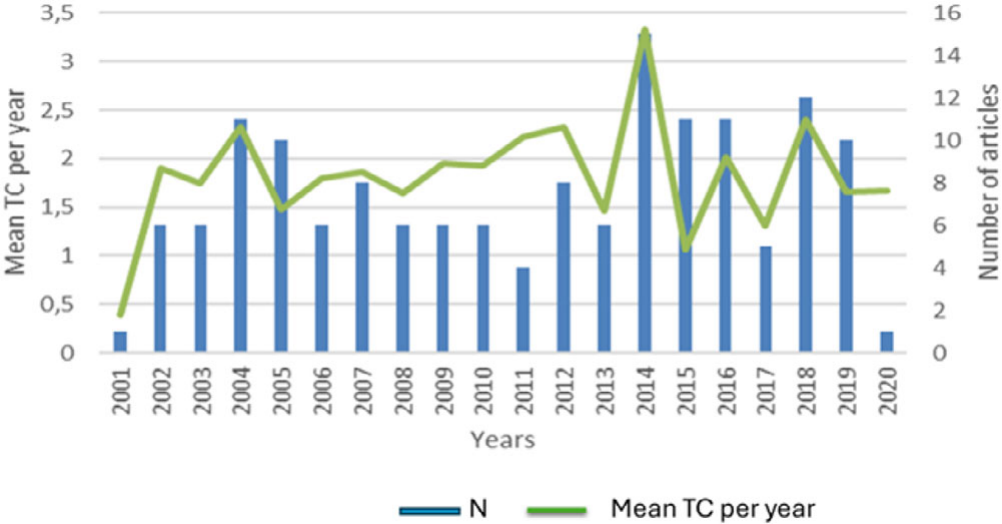
Publication and citation per year. TC, total citation.

Figure 2 illustrates the developmental trajectory, the evolution of academic interest and the annual citation trends related to the MHCI gene in agricultural livestock. Significantly, the Year 2014 emerged as the only year with the greatest number of publications (*n* = 15) and the highest annual citation total of 3, with the Years 2018 and 2004 producing between 2 and 3 citations for studies concerning the MHCI gene in farm animals.

### 3.3 Most Relevant Authors

**FIGURE 3:**
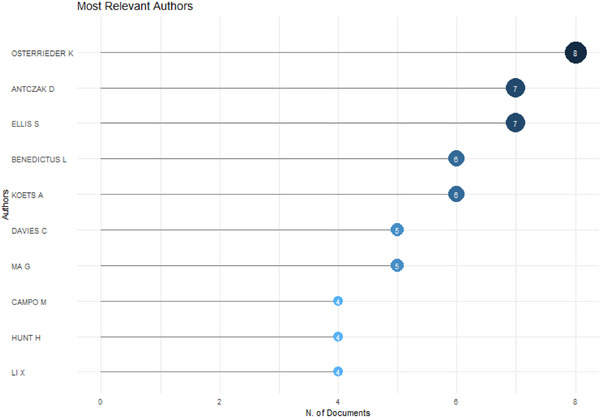
Most relevant authors (top 10).

The results revealed that within the top 10 leading researchers in the specialised area of MHCI gene in livestock, Osterrieder K. stood out as the primary contributor, having produced eight publications (*n* = 8). This was followed by Antczak D., and Ellis S., each with seven (*n* = 7) publications. At the lower end of the spectrum, Compo M., Hunt M. and Li X. each contributed four documents, as depicted in Figure 3.

### 3.4 Authors Production Over Time

**FIGURE 4:**
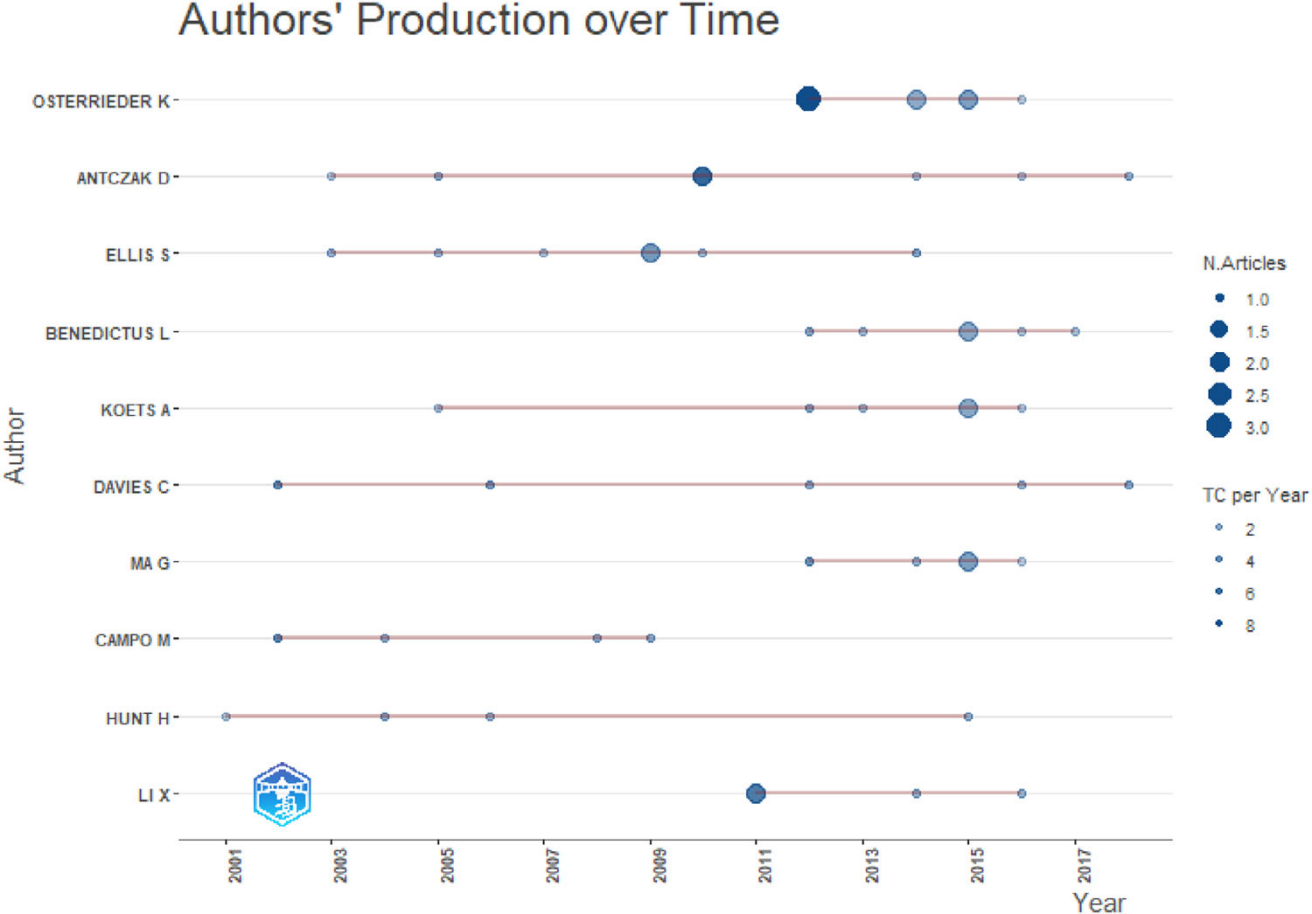
Authors production over time.

Figure 4 illustrates the contributions of the authors over time; the work of Ellis S. was consistently cited from 2003 to 2014. Hunt H. was the sole author whose work was cited in 2001. The authors Osterrieder K., Antczak D., Benedictus L., Koets A., Davies C., Ma G. and Li X. were cited from 2016 to 2019.

### 3.5 Production of Countries Over Time

**FIGURE 5:**
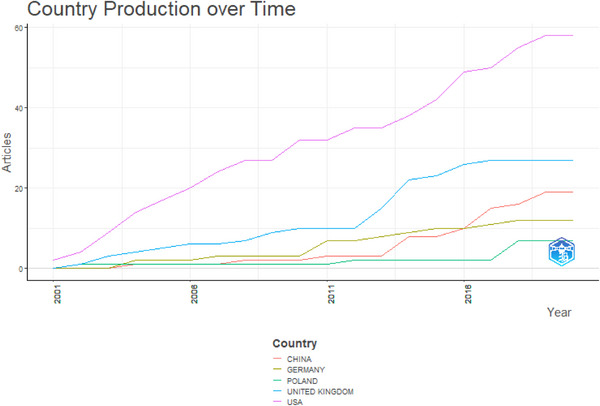
Country production over time.

Figure 5 depicts the production output of different countries from 2001 to 2020. Beginning in 2001, the United States of America was at the forefront with an initial production of 2 articles (*n* = 2), maintaining a consistent output until 2020, when it reached a peak of 58 articles (*n* = 58). The United Kingdom, which started in 2001 with no articles produced (*n* = 0), saw substantial growth from 2002 to 2020, culminating in a peak production of 27 articles (*n* = 27). China initiated its article production in 2004, achieving a peak of 19 articles (*n* = 19) by 2020. Germany also began its production between 2004 and 2020, with a maximum output of 12 articles (*n* = 12). Both China and Poland started their production activities during the period from 2001 to 2020, each reaching a peak of 7 articles.

### 3.6 Most Relevant Affiliations

**FIGURE 6:**
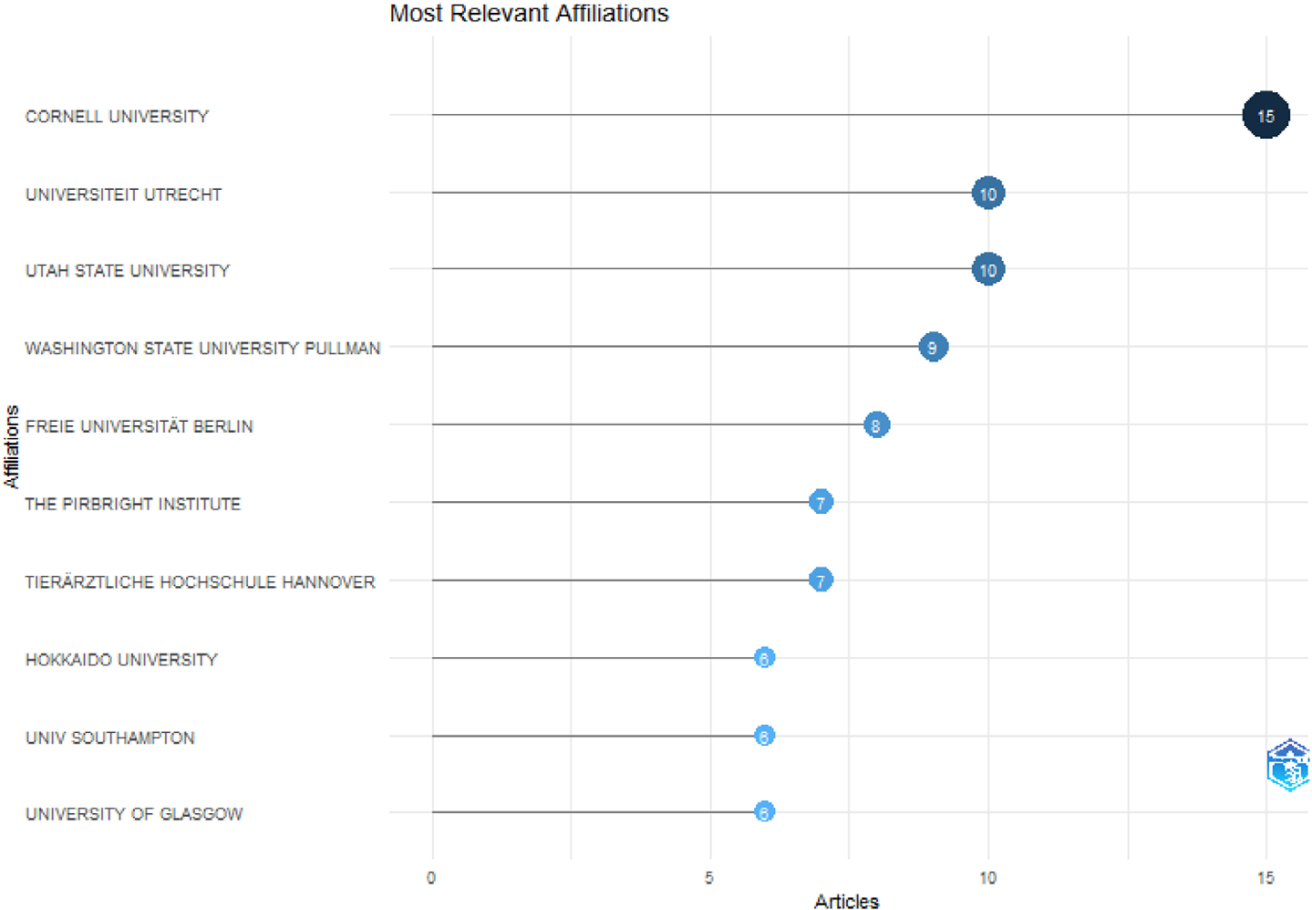
Most relevant affiliations (top 10).

The top 10 affiliations ranked by the number of published articles are depicted in Figure 6. The analysis revealed that Cornell University in the United States contributed a total of 15 articles (*n* = 15) to this area of research. Following this, two institutions, the Universiteit Utrecht in the Netherlands and Utah State University in the United States, each produced 10 articles (*n* = 10). At the lower end, with 6 articles (*n* = 6), were Hokkaido University in Japan, the University of Southampton and the University of Glasgow, all situated in the United Kingdom.

### 3.7 Most Global Cited Article

The articles globally cited during the designated period of this research are detailed in Table 2. The document by Sivakumar T., released in 2014 in the *Infection, Genetics and Evolution Journal*, achieved the highest citation count, totalling 198, which corresponds to an average of 16.5 citations annually throughout the study's timeframe. Following this, the research conducted by Yuan T., published in 2014 in the *Journal of Biomedical Materials Research Part B: Applied Biomaterials*, accumulated 133 citations, averaging 11.08 citations per year.

**TABLE 2:** Most globally cited article.

Author, year and journal	Total citation	Total citations per year	Article title
Sivakumar T., 2014, *Infection, Genetics and Evolution*	198	16.5	Evolution and Genetic Diversity of Theileria
Yuan T., 2014, *Journal of Biomedical Materials Research: Part B Applied Biomaterials*	133	11.08	Collagen Hydrogel as an Immunomodulatory Scaffold in Cartilage Tissue Engineering
Naslavsky N., 2004, *Molecular Biology of the Cell*	120	5.45	Rabenosyn‐5 and EHD1 Interact and Sequentially Regulate Protein Recycling to the Plasma Membrane
Morein B., 2004, *Advanced Drug Delivery Reviews*	116	5.27	Current Status and Potential Application of ISCOMs in Veterinary Medicine
Granados D., 2009, *BMC Immunology*	112	6.59	ER Stress Affects Processing of MHC Class I‐Associated Peptides
Hill J., 2002, *Biology of Reproduction*	99	4.13	Abnormal Expression of Trophoblast Major Histocompatibility Complex Class I Antigens in Cloned Bovine Pregnancies Is Associated with a Pronounced Endometrial Lymphocytic Response
Nene V., 2016, *Ticks and Tick‐Borne Diseases*	96	9.6	The Biology of *Theileria parva* and Control of East Coast Fever—Current Status and Future Trends
Ashrafi G., 2002, *Oncogene*	95	3.96	Down‐Regulation of MHC Class I by Bovine Papillomavirus E5 Oncoproteins
Mollenkopf H., 2004, *Infection and Immunity*	88	4	Application of Mycobacterial Proteomics to Vaccine Design: Improved Protection by *Mycobacterium bovis* BCG Prime–Rv3407 DNA Boost Vaccination Against Tuberculosis
Flierman D., 2003, *Journal Biological Chemistry*	86	3.74	Polyubiquitin Serves as a Recognition Signal, Rather than a Ratcheting Molecule, During Retro‐Translocation of Proteins Across the Endoplasmic Reticulum Membrane

### 3.8 Word Cloud Keywords

**FIGURE 7:**
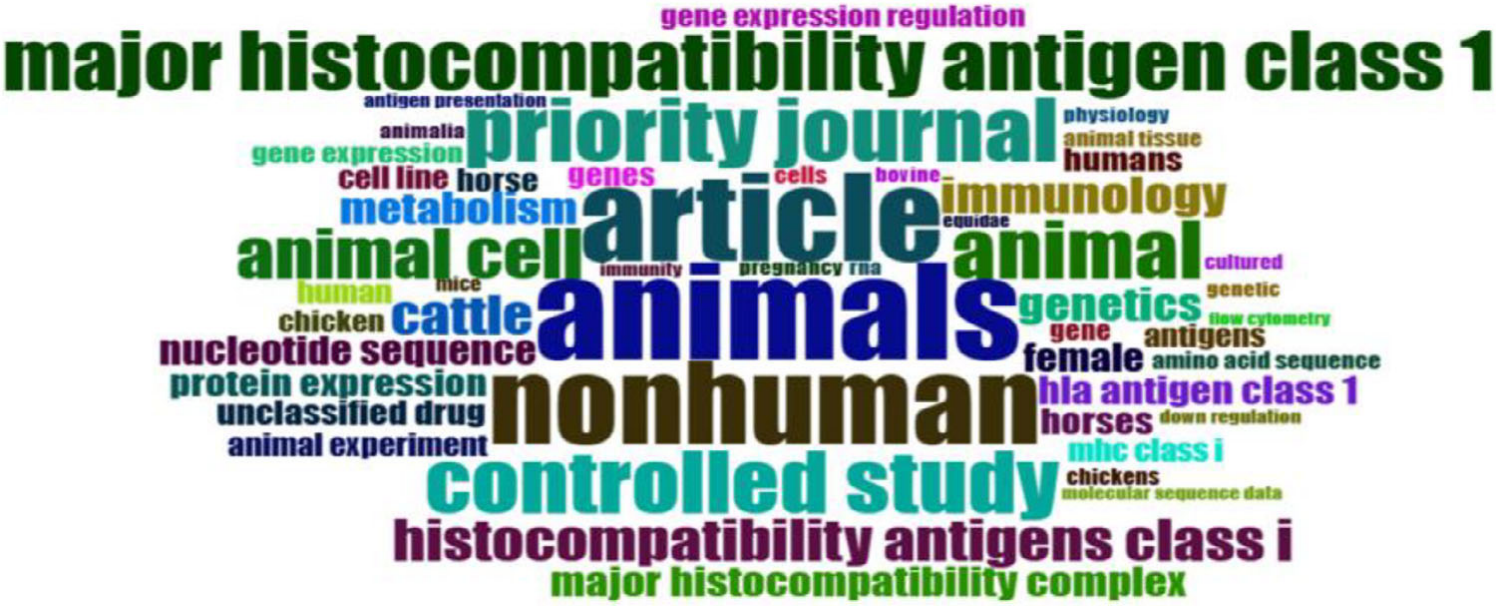
Word cloud keywords.

Figure 7 illustrates the most commonly utilised terms within the analysed articles. The term ‘animals’ was highlighted in a more vivid colour and presented in a bolder font, signifying its significance and prevalence across the majority of the articles pertaining to the topic, ‘MHCI gene in farm animals.’ This was followed by the terms ‘major histocompatibility antigen Class I’ and ‘non‐human.’ In contrast, the term ‘flow cytometry’ appeared to be infrequently employed.

### 3.9 Trending Topics

**FIGURE 8:**
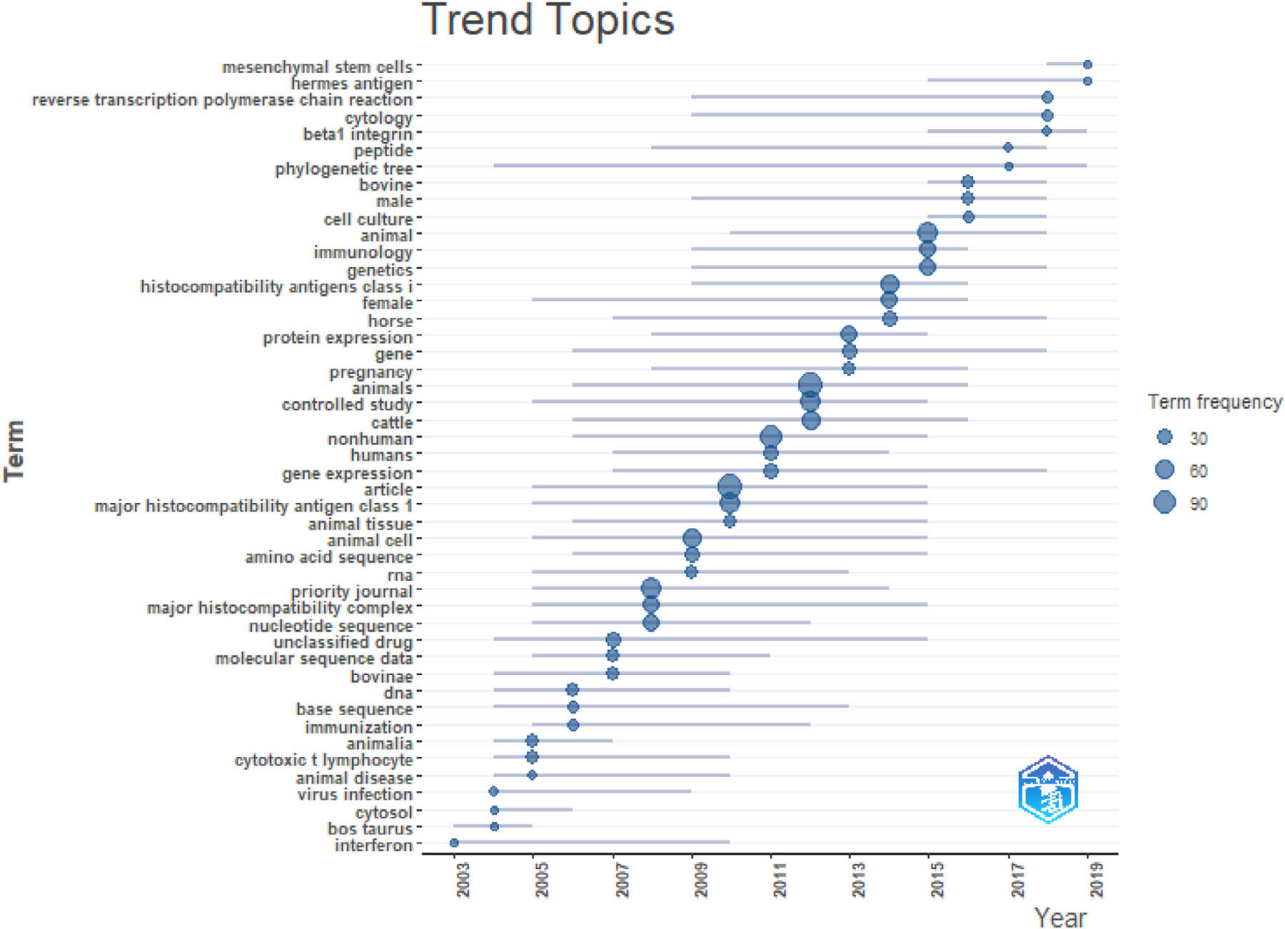
Trending topics.

Figure 8 shows the emerging research themes related to MHCI, concerning farm animals. The designation ‘Animals’ represents the most frequently occurring term, with a total frequency of 118 during the period from 2006 to 2016. In contrast, the term ‘Major Histocompatibility antigen Class I’ recorded a frequency of 73 from 2005 to 2015. Furthermore, between 2015 and 2019, subjects associated with the term ‘bovine’ exhibited a notably low frequency of 19.

### 3.10 Thematic Map

**FIGURE 9:**
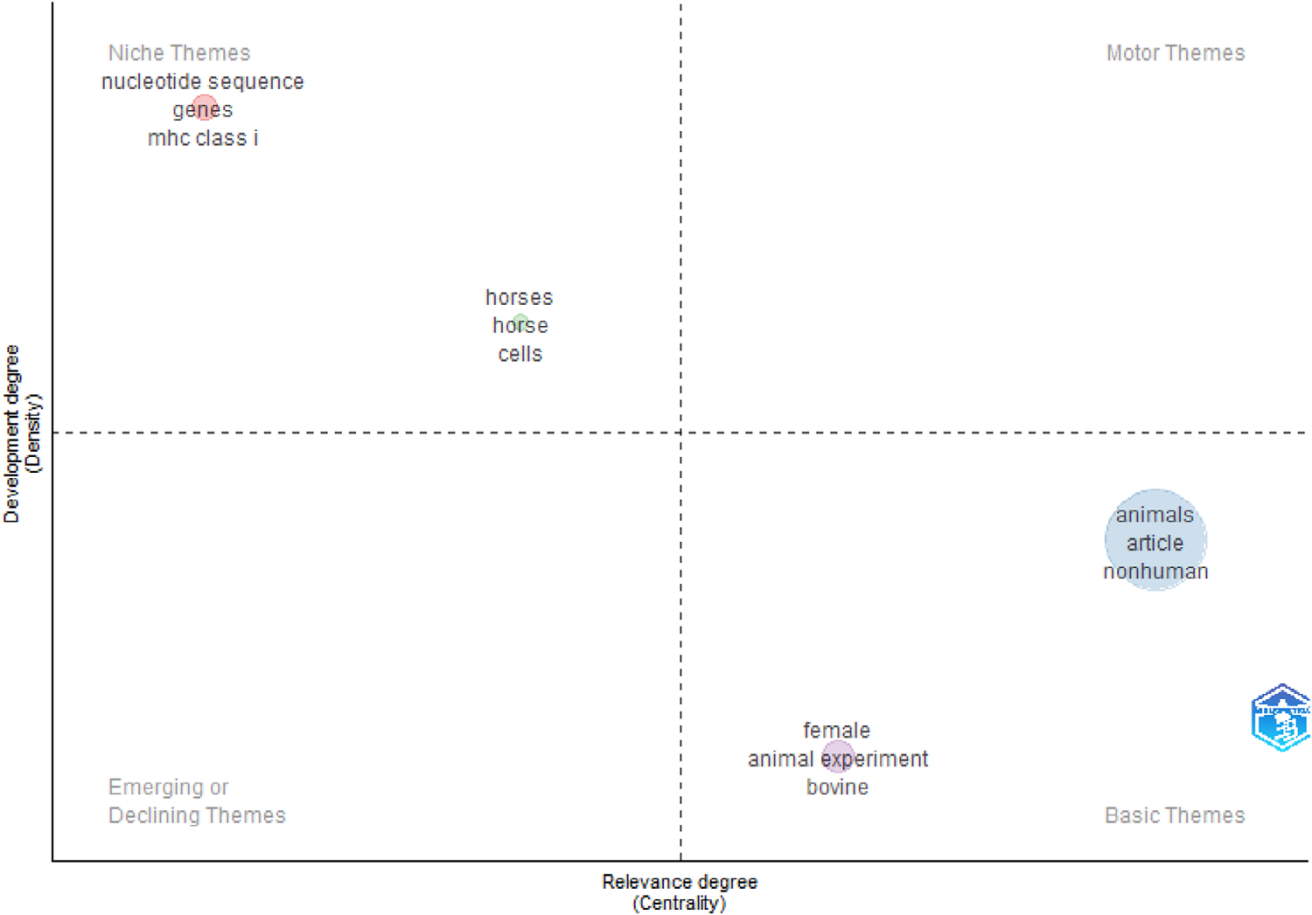
Thermatic map.

A thematic map illustrating frequently occurring keywords related to the MHCI gene in research on farm animals, derived from author keywords, is presented in Figure 9 The analysis identified four clusters, each of which contained the top 12 keywords. The upper left quadrant, which encompasses niche themes, is characterised by low centrality and a significantly high density. Topics that fall into this quadrant, ‘Nucleotide sequence, genes, MHC Class I’, are extremely detailed, underrepresented and changing quickly. Also within this quadrant were topics ‘horse, horse and cells’ with high density and high centrality; these topics were high impact but with no specialisation. Topics with low impact and high specialisation were found in the lower right quadrant, those with slightly high density and significantly high centrality involved ‘animals, article and non‐human’ while those with a significantly high centrality and low density involved ‘female, animal experiment and bovine’

### 3.11 Collaboration Network

**FIGURE 10:**
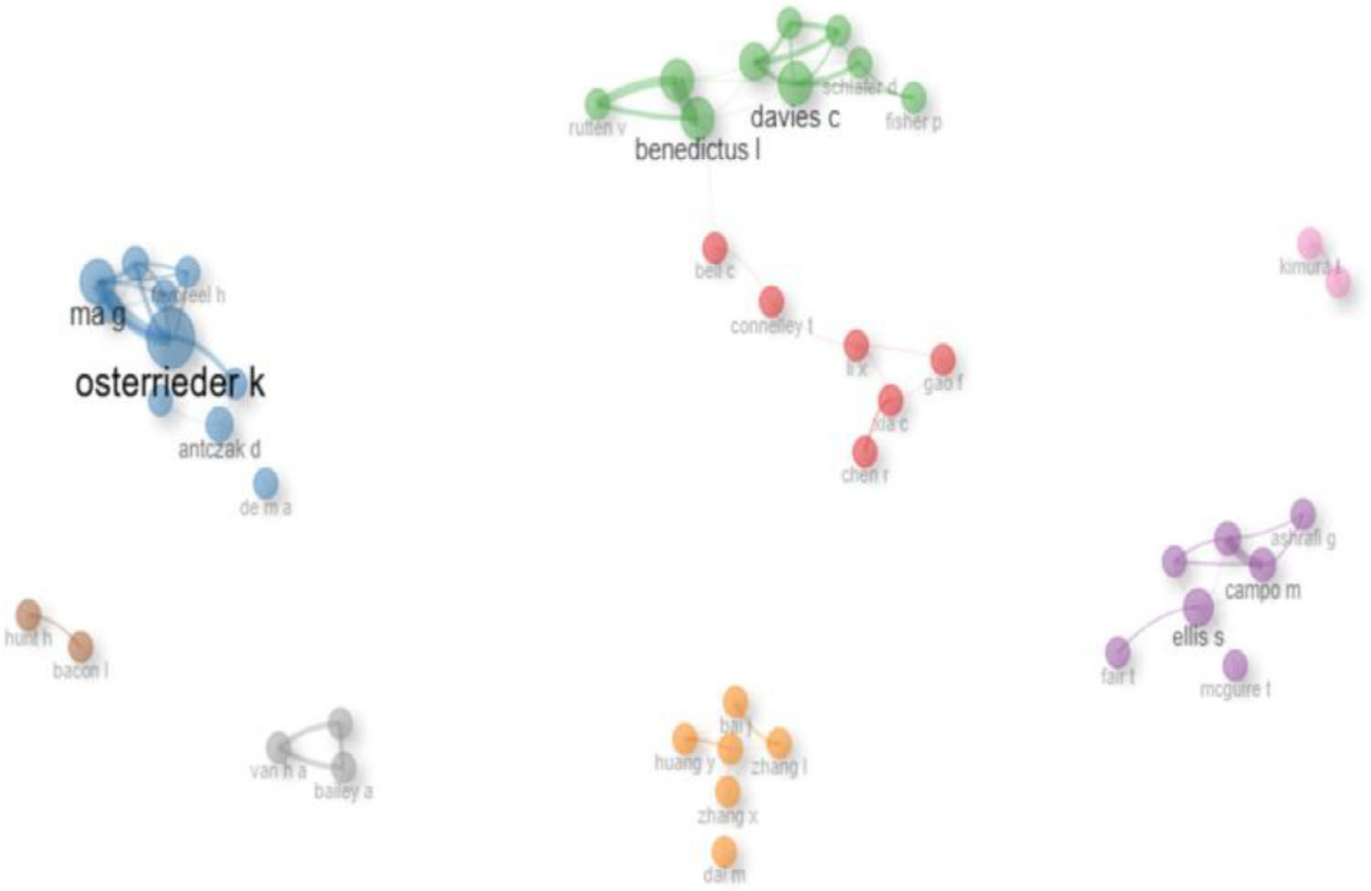
Co‐authorship network.

Figure 10 shows the co‐authorship network for research on the MHCI gene in research on farm animals. The author network consists of eight unique clusters that demonstrate the level of collaboration among various research groups. The connections between the green, red, blue and purple clusters indicate that the authors in these groups often participate in collaborative activities. Author Benedictus L. exhibited the highest number of collaborations both within the same cluster and across different clusters, whereas authors Bailey A. and Van HA recorded the fewest collaborations.

### 3.12 Countries Collaboration World Map

Table 3 illustrate the nations that engaged in collaborative efforts during the specified timeframe analysed in this study. The findings revealed that numerous countries engaged in collaboration. Nevertheless, the USA and Korea exhibited the greatest number of collaborative efforts (*n* = 127). This was followed by Korea and Vietnam, with both the USA and Vietnam demonstrating collaborations at a frequency of one hundred and six (*n* = 106). Collaborations with negative frequencies contributed more to the collaborations than receiving.

**TABLE 3:** Countries collaboration world map.

From	To	Frequency
Ireland	Kenya	37
Korea	Vietnam	106
Spain	Switzerland	8
United Kingdom	Ireland	−8
United Kingdom	Isreal	35
United Kingdom	Kenya	37
United States of America	Ireland	−8
United States of America	Isreal	35
United States of America	Kenya	37
United States of America	Korea	127
United States of America	United Kingdom	−2
United States of America	Vietnam	106

## 4 Discussion

The MHCI is responsible for adaptive immune response against pathogens, where it presents itself by regulating the natural killer cell function and is composed of several structural domains that are active in peptide binding (Zidi et al. 2008). The objective of this study was to uncover emerging trends related to the MHCI gene in farm animals using bibliometric analysis approach. According to the research criteria applied in this study, bibliometric analysis was carried out in 149 articles between 2001 and 2020, focusing on the topic, ‘MHCI gene in farm animals.’ The researcher who has produced the highest number of documents during the designated timeframe in this study has concentrated on subjects pertaining to MHCI in pigs, horses and chickens, examining viral entry and the mechanisms of immune evasion (Osterrieder et al. 2003; Tavlarides‐Hontz et al. 2009; Said et al. 2011; Huang et al. 2014; Haung and Osterrieder 2015).

The publisher with the second greatest number of documents produced engaged in research concerning cattle immunology and killer immunoglobulin‐like receptors (Ellis and Hammond 2014; Allan et al. 2015; Ballingall et al. 2018; Gibson et al. 2020). The article that received the most citations globally investigated the evolution and genetic diversity of a parasite impacting ruminants (Sivakumar et al. 2014). Benedictus Lindert holds the record for the highest number of collaborations, attributed to his work focused on identifying solutions related to the genetic resistance of animals and developing vaccines to combat fatal diseases in the bovine sector (Benedictus et al. 2012; Benedictus 2015, 2016; Hoekstra et al. 2018; Benedictus et al. 2019; Callaby et al. 2020). Although bibliometric analysis has been conducted in animal science, there have been no studies on MHCI gene in farm animals, making this the first study. The production output of articles over time showed that the United States of America and the United Kingdom were found to be leading in this regard. The findings were somewhat comparable to those reported by Zhou et al. (2022), who similarly recognised the United States as a prominent contributor to research concerning bibliometric analysis of smart livestock from 1998–2022.

The United States possesses substantial resources, enabling it to undertake genetic studies on livestock, which tend to be costly and necessitate specialised equipment typically available only in fully developed nations (Marino et al. 2023). According to National Institute of Food and Agriculture (NIFA) (2025), the United Kingdom and the United States of America have established a collaborative agreement aimed at promoting research of significant strategic importance to both nations, particularly in the domain of Animal Health & Disease, which encompasses Veterinary Immune Reagents. Through this initiative, the Biotechnology and Biological Sciences Research Council (BBSRC) and National Institute of Food and Agriculture (NIFA) have jointly financed five collaborative projects between the United States and the United Kingdom, focusing on controlling the dissemination of pathogens and reducing health risks as well as environmental consequences associated with global food production (UKRI 2020).

This partnership could elucidate the reason these two nations (the United Kingdom and the United States of America) are not only at the forefront of research output concerning the MHCI gene in livestock, but are also the primary nations in establishing collaborations with other countries. The results of Marino et al. (2023) on ‘Scientific Productions on Precision Livestock Farming: An Overview of the Evolution and Current State of Research Based on a Bibliometric Analysis,’ which identified one of the most dominant livestock species to be cattle. This aligns with the findings of this study; the researchers' interests were focused on the bovine industry, which includes both dairy and beef cattle. The worldwide consumption of animal products exhibits a significant demand for beef, alongside other items such as milk and cheese, which are derived from these animals (Miller et al. 2022). According to Rasmussen et al. (2024), the annual economic losses incurred in dairy cattle because of diseases amount to approximately US $ 65 billion.

This may be a contributing factor motivating researchers to concentrate on bovines, aiming to align their projects with industry interests to secure funding. The role of farm animals, particularly small livestock such as goats and sheep, is essential for food security; however, there has been a notable lack of research focused on the MHCI gene in these species. This gap has resulted in a limited body of literature on the topic. Africa ranks second to Asia regarding the population of goats; however, the goats found on these continents are predominantly indigenous and are typically owned by impoverished individuals, not for commercial gain, but primarily for essential social advantages (Utaaker et al. 2021). In spite of a steadily rising global demand for goat meat, it continues to be less than that for cattle (Palmer et al. 2022). Consequently, this restricts researchers from advancing towards studies that concentrate on molecular work, particularly those associated with goats, as a thorough comprehension of the MHCI gene incurs significant costs.

Market demand plays a critical role towards informing funding interest, especially in developing countries. Despite this, goats and sheep have garnered interest from researchers in various other fields, indicating their potential to enhance the volume of scholarly work in this area. Furthermore, the global farming of these animals presents opportunities for broader collaborative efforts in research. The pre‐eminence of highly developed nations in the generation of scientific literature concerning the MHCI gene in livestock may result in a constrained global perspective on the trends within this domain.

## 5 Conclusion

Research trends concerning the MHCI gene in livestock have indicated a significant focus on understanding disease infiltration into the animals' immune systems and exploring strategies to manage this invasion to prevent harm to the animals. The thematic map shows that the integration of impactful terms like MHC Class I with specialised terms, such as animals in future discussions will attract interest in upcoming research within the field. The findings of this study clearly indicate that Africa and Asia, being continents with significant goat populations, should initiate collaborative efforts towards research involving MHCI in goats, particularly in meat goat breeds.

## Author Contributions

**Masixole Maswana, Dikeledi Petunia Malatji and Thobela Loius Tyasi**: conceptualisation, funding acquisition, writing. **Masixole Maswana**: data curation, formal analysis, investigation, writing. **Masixole Maswana and Thobela Loius Tyasi**: methodology, project administration, resources, software. **Dikeledi Petunia Malatji and Thobela Loius Tyasi**: supervision, validation.

## Funding

The authors have nothing to report.

## Ethics Statement

This research utilised a dataset that is publicly accessible from the Scopus (Elsevier.s data and Web of Science (WoS), thus eliminating the need for ethical considerations.

## Conflicts of Interest

The authors declare no conflicts of interest.

## Data Availability

Data was obtained from Scopus (Elsevier) and Web of Science.

## References

[vms370818-bib-0001] Allan, A. , N. Sanderson , S. Gubbins , S. Ellis , and J. Hammond . 2015. “Cattle NK Cell Heterogeneity and the Influence of MHC Class I.” Journal of Immunology 195: 2199–2206. 10.4049/jimmunol.1500227. PMC4543905 26216890

[vms370818-bib-0002] Baas, J. , M. Schotten , A. Plume , G. Côté , and R. Karimi . 2020. “Scopus as a Curated, High‐Quality Bibliometric Data Source for Academic Research in Quantitative Science Studies.” Quantitative Science Studies 1: 377–386. 10.1162/qss_a_00019.

[vms370818-bib-0003] Ballingall, K. , R. Bontrop , S. Ellis , et al. 2018. “Comparative MHC Nomenclature: Report From the ISAG/IUIS‐VIC Committee.” Immunogenetics 70: 625–632. 10.1007/s00251-018-1073-3. 30039257

[vms370818-bib-0004] Banchi, P. , A. Rota , A. Bertero , et al. 2022. “Trends in Small Animal Reproduction: A Bibliometric Analysis of the Literature.” Animals 12, no. 3: 336. 35158661 10.3390/ani12030336 PMC8833461

[vms370818-bib-0005] Benedictus, L. 2015. “Bovine Materno‐Fetal Alloimmune Mediated Disorders: MHC Class I (in) Compatibility in Retained Fetal Membranes and Bovine Neonatal Pancytopenia.” Doctoral thesis, Universiteit Utrecht. Uitgeverij BOXPress. https://dspace.library.uu.nl/handle/1874/310576.

[vms370818-bib-0006] Benedictus, L. , L. Ravesloot , K. Poppe , et al. 2019. “Immunization of Young Heifers With Staphylococcal Immune Evasion Proteins Before Natural Exposure to *Staphylococcus aureus* Induces a Humoral Immune Response in Serum and Milk.” BMC Veterinary Research 15: 15. 10.1186/s12917-018-1765-9. 30616609 PMC6323680

[vms370818-bib-0007] Benedictus, L. , V. P. M. G. Rutten , and A. P. Koets . 2016. “Pregnancy Boosts Vaccine‐Induced Bovine Neonatal Pancytopenia‐Associated Alloantibodies.” Vaccine 34, no. 8: 1002–1005. 10.1016/j.vaccine.2016.01.013. 26796141

[vms370818-bib-0008] Benedictus, L. , A. J. Thomas , R. Jorritsma , C. J. Davies , and A. P. Koets . 2012. “Two‐Way Calf to Dam Major Histocompatibility Class I Compatibility Increases Risk for Retained Placenta in Cattle.” American Journal of Reproductive Immunology 67, no. 6: 179–183. 10.1111/j.1600-0897.2011.01085.x. 22035222

[vms370818-bib-0009] Callaby, R. , R. Kelly , S. Mazeri , et al. 2020. “Genetic Diversity of Cameroon Cattle and a Putative Genomic Map for Resistance to Bovine Tuberculosis.” Frontiers in Genetics 11: 550215. 10.3389/fgene.2020.550215. 33281865 PMC7705233

[vms370818-bib-0010] Çelik, Ş. 2021. “The Bibliometric Analysis of the Studies Conducted in the Field of Water Buffalo Breeding.” Progress in Nutrition 23, no. 2:.

[vms370818-bib-0011] Cui, L. , W. Tang , X. Deng , and B. Jiang . 2023. “Farm Animal Welfare Is a Field of Interest in China: A Bibliometric Analysis Based on Cite Space.” Animals 13, no. 19: 3143. 37835750 10.3390/ani13193143 PMC10571665

[vms370818-bib-0012] Ellis, S. A. , and J. A. Hammond . 2014. “The Functional Significance of Cattle Major Histocompatibility Complex Class I Genetic Diversity.” Annual Review of Animal Biosciences 2, no. 1: 285–306. 25384144 10.1146/annurev-animal-022513-114234

[vms370818-bib-0013] Gibson, M. S. , A. J. Allan , N. Sanderson , et al. 2020. “Two Lineages of KLRA With Contrasting Transcription Patterns Have Been Conserved at a Single Locus During Ruminant Speciation.” Journal of Immunology 204, no. 9: 2455–2463. 10.4049/jimmunol.1801363. PMC7167460 32213565

[vms370818-bib-0014] Grossen, G. , L. Keller , I. Iris Biebach , and D. Croll , The International Goat Genome Consortium . 2014. “Introgression From Domestic Goat Generated Variation at the Major Histocompatibility Complex of Alpine Ibex.” PLOS Genetics 10, no. 6: e1004438. 10.1371/journal.pgen.1004438. 24945814 PMC4063738

[vms370818-bib-0015] Hoekstra, J. , V. Rutten , L. Sommeling , et al. 2018. “High Production of LukMF' in *Staphylococcus aureus* Field Strains Is Associated With Clinical Bovine Mastitis.” Toxins 10, no. 5: 200. 10.3390/toxins10050200. 29762488 PMC5983256

[vms370818-bib-0016] Huang, T. , M. J. Lehmann , A. Said , G. Ma , and N. Osterrieder . 2014. “Major Histocompatibility Complex Class I Downregulation Induced by Equine Herpesvirus Type 1 pUL56 Is Through Dynamin‐Dependent Endocytosis.” Journal of Virology 88, no. 21: 12802–12815. 10.1128/JVI.02079-14. 25165105 PMC4248885

[vms370818-bib-0017] Huang, T. , G. Ma , and N. Osterrieder . 2015. “Equine Herpesvirus 1 Multiply Inserted Transmembrane Protein pUL43 Cooperates With pUL56 in Downregulation of Cell Surface Major Histocompatibility Complex Class I.” Journal of Virology 89, no. 12: 6251–6263. 10.1128/JVI.00032-15. 25833055 PMC4474290

[vms370818-bib-0018] Marino, R. , P. Francesca , and A. Fabio . 2023. “Scientific Productions on Precision Livestock Farming: An Overview of the Evolution and Current State of Research Based on a Bibliometric Analysis.” Animals 13, no. 14: 2280. 37508057 10.3390/ani13142280 PMC10376211

[vms370818-bib-0019] Miller, V. , J. Reedy , F. Cudhea , et al. and Global Dietary Database . 2022. “Global, Regional, and National Consumption of Animal‐Source Foods Between 1990 and 2018: Findings From the Global Dietary Database.” Lancet Planetary Health 6, no. 3: e243–e256. 10.1016/S2542-5196(21)00352-1. 35278390 PMC8926870

[vms370818-bib-0020] National Institute of Food and Agriculture (NIFA) . 2025. United States Department of Agriculture. nifa.usda.gov/data.

[vms370818-bib-0021] Osterrieder, N. , D. Schumacher , S. Trapp , M. Beer , J. Einem , and K. Tischer . 2003. “Establishment and Use of Infectious Bacterial Artificial Chromosome (BAC) DNA Clones of Animal Herpesviruses.” Berliner Und Munchener Tierarztliche Wochenschrift 116: 373–380. https://www.ncbi.nlm.nih.gov/pubmed/14526467. 14526467

[vms370818-bib-0022] Palmer, k. , A. Naicker , and U. Kolanisi . 2022. “The Potential of Goat Meat Acceptance by Young Adults in South Africa.” African Journal of Inter/Multidisciplinary 4, no. 1: 406–418.

[vms370818-bib-0023] Rasmussen, P. , H. W. Barkema , P. P. Osei , et al. 2024. “Global Losses due to Dairy Cattle Diseases: A Comorbidity‐Adjusted Economic Analysis.” Journal of Dairy Science 107, no. 9: 6945–6970. 38788837 10.3168/jds.2023-24626 PMC11382338

[vms370818-bib-0024] Ribeiro, S. A. , C. B. Marins de Campos , H. S. Gonçalves de Sousa , A. Silva da Cruz , and A. Divino da Cruz . 2021. “Suitability of Scienciometric Analysis Targeting Loop Mediated Isothermal Amplification Assay Applied to Farm Animals.” Brazilian Journal of Development 7, no. 12: 115333–115354.

[vms370818-bib-0025] Said, A. , A. Damiani , G. Ma , D. Kalthoff , M. Beer , and N. Osterrieder . 2011. “An Equine Herpesvirus 1 (EHV‐1) Vectored H1 Vaccine Protects Against Challenge With Swine‐Origin Influenza Virus H1N1.” Veterinary Microbiology 154, no. 1: 113–123. 10.1016/J.VETMIC.2011.07.003. 21803510

[vms370818-bib-0026] Sivakumar, T. , K. Hayashida , C. Sugimoto , and N. Yokoyama . 2014. “Evolution and Genetic Diversity of Theileria.” Infection, Genetics and Evolution 27: 250–263. 10.1016/j.meegid.2014.07.013. 25102031

[vms370818-bib-0027] Tavlarides‐Hontz, P. , P. M. Kumar , J. R. Amortegui , N. Osterrieder , and M. S. Parcells . 2009. “A Deletion Within Glycoprotein L of Marek's Disease Virus (MDV) Field Isolates Correlates With a Decrease in Bivalent mdv Vaccine Efficacy in Contact‐Exposed Chickens.” Avian Diseases 53, no. 2: 287–296. 19630238 10.1637/8558-121208-Reg.1

[vms370818-bib-0028] Tyasi, T. L. , M. Ergin , and M. C. Mathapo . 2024. “A Bibliometric Analysis of the Literature on Goat Breeding.” F1000Research 13: 451.

[vms370818-bib-0029] United Kingdom Research and Innovation (UKRI) . 2020. The Impact of UK‐US Research Collaboration. UKRI. www.ukri.org.

[vms370818-bib-0030] Utaaker, K. S. , S. Chaudhary , T. Kifleyohannes , and L. J. Robertson . 2021. “Global Goat! Is the Expanding Goat Population an Important Reservoir of Cryptosporidium?” Frontiers Veterinary Sciences 8: 648500. 10.3389/fvets.2021.648500. PMC7977713 33748221

[vms370818-bib-0031] Zhou, Y. , W. Tiemuer , and L. Zhou . 2022. “Bibliometric Analysis of Smart Livestock From 1998–2022.” Procedia Computer Science 214: 1428–1435.

[vms370818-bib-0032] Zidi, A. , A. Sa`nchez , G. Obexer‐Ruff , and M. Amills . 2008. “Sequence Analysis of Goat Major Histocompatibility Complex Class I Genes.” Journal of Dairy Science 91, no. 2: 814–817. 18218769 10.3168/jds.2007-0342

